# The Investor Psychology and Stock Market Behavior During the Initial Era of COVID-19: A Study of China, Japan, and the United States

**DOI:** 10.3389/fpsyg.2021.626934

**Published:** 2021-02-10

**Authors:** Sobia Naseem, Muhammad Mohsin, Wang Hui, Geng Liyan, Kun Penglai

**Affiliations:** ^1^School of Economics and Management, Shijiazhuang Tiedao University, Shijiazhuang, China; ^2^School of Business, Hunan University of Humanities, Science and Technology, Loudi, China

**Keywords:** COVID-19, investor psychology, stock market behavior, financial sustainability, masses psychology

## Abstract

A highly transmittable and pathogenic viral infection, COVID-19, has dramatically changed the world with a tragically large number of human lives being lost. The epidemic has created psychological resilience and unbearable psychological pressure among patients and health professionals. The objective of this study is to analyze investor psychology and stock market behavior during COVID-19. The psychological behavior of investors, whether positive or negative, toward the stock market can change the picture of the economy. This research explores Shanghai, Nikkei 225, and Dow Jones stock markets from January 20, 2020, to April 27, 2020, by employing principal component analysis. The results showed that investor psychology was negatively related to three selected stock markets under psychological resilience and pandemic pressure. The negative emotions and pessimism urge investors to cease financial investment in the stock market, and consequently, the stock market returns decreased. In a deadly pandemic, the masses were more concerned about their lives and livelihood and less about wealth and leisure. This research contributes to the literature gap of investors’ psychological behavior during a pandemic outbreak. The study suggests that policy-makers should design a plan to fight against COVID-19. The government should manage the health sector’s budget to overcome future crises.

## Introduction

The terminology of “Corona” is not newly invented in science. This single-stranded RNA virus’ primary roots were observed in 1960, belonging to the Corona viridae family in the order Nidovirales (Galante et al., 2016; Kanwar et al., 2017; Guo et al., 2020; Mohsin et al., 2020b). The taxonomic naming comes from the virus’ structure, which gives the appearance of crown-like spikes on the virus’ outer surface (Azam et al., 2020; Sarfraz et al., 2020c; Shereen et al., 2020). The prey of the first coronavirus species was chicken and pig; there was no human–human transmission. From 1960 to 2020, different allied versions of the same family of viruses have been observed: the common cold in adults (COV 229E and COV OC43 in mid-1960); severe acute respiratory syndrome coronavirus (SARS-CoV-2003); human coronavirus with common cold, bronchitis, and asthma; chronic obstructive pulmonary disease (COPD) exacerbations; pneumonia (HCOV NL63-2004 and CoV-HKU1); Middle East respiratory syndrome (MERS CoV-2012); and severe acute respiratory syndrome coronavirus-19 (SARS-CoV-2019 or SARS-CoV-2), displaying unmatched intensity and severity compared to the previous species of corona (Van Der Hoek et al., 2004; Kahn and McIntosh, 2005; Woo et al., 2005; Esper et al., 2006; Zaki et al., 2012). At the start of the virus breakout, the virus name was 2019-nCOV as per the International Committee on Taxonomy of Viruses (ICTV), and the Chinese Center for Disease Control and Prevention (CCDC) changed it into SARS-CoV-2 on January 7, 2020 due to its structure and symptoms. COVID-19 was first discovered in Wuhan’s wet market, Hubei Province, China, in early December 2019, and this aroused global attention in late January 2020. The virus has been spreading exponentially, using human-to-human transmission through respiratory droplets, i.e., sneezing and coughing (Azam et al., 2020; Li et al., 2020; Sarfraz et al., 2020a; Shereen et al., 2020). During this incubation period, researchers focused on exploring, preventing, and treating patients. Still, the pandemic’s psychological impact is the other side of the disease (mental illness). The global quarantine announcement has sparked several concerns: fear of separation from family, fear of illness and death, avoidance of medical facilities due to threat of infection, fear of unemployment, the threat of racism against people who live in or are perceived to be from the affected areas, fear of losing near and dear ones because of the virus, maintained space from minors and disabled or elderly family members due to infection, isolation, and recalling the severity of the treatment of infected people. These have become originators of anxiety, stress, and grave concern globally. These mental health aspects of the COVID-19 outbreak have affected individual lives as well as the financial markets.

### Human Psychology and COVID-19

The current pandemic of SARS-CoV-2 has seriously influenced human psychology through a notable mental state of “anxiety.” The term “anxiety” covers the population’s reaction toward the epidemic to all media, whether the information is authentic or erroneous, e.g., inappropriate behavior of people concerning the abandonment of animals and panic buying of other foods. The panic attacks are not properly defined without linkage to anxiety disorder in the medical sense. Anxiety is a combination of different psychiatric disorders both internal (phobias, panic attacks, and panic disorder) and external (worry, stress, fear, painful experiences, or events). The psychological effect of COVID-19’ has led to mass hysteria, post-traumatic stress disorder (PTSD), panic attacks, obsessive-compulsive disorder (OCD), and generalized anxiety disorder (GAD). The behavioral immune system (BIS) theory, stress theory, and perceived risk theory explain that negative emotion (anxiety, aversion) and negative cognitive assessment of human beings are developed for self-protection. People tend to develop avoidant behavior and strictly follow the social norms due to the pandemic’s severe effects and the potential threat of disease (Cao et al., 2020; Lai et al., 2020; Sarfraz et al., 2020b). The anxiety, stress, and panic attacks of people due to COVID-19 have created two etiologies. The first is the identification of symptoms of acute respiratory distress syndrome (ARDS), such as cough and dyspnea, at high frequency (Preter and Klein, 2008; Javelot and Weiner, 2020). The second one is “false alarming” (Klein, 1993) as a psychopathological link to the catastrophic interpretation of physiological sensation (respiration rate). The recurrence of panic attacks has increased the respiration rate and has become the reason for excessively avoidant behaviors and blind conformity (Li et al., 2020; Mohsin et al., 2020a). Psychopathology is a keen concern for this study because it has an intense effect on investor behavior. Stock market investors and business people generally spend most of their time in the workplace. However, they are currently mostly homebound; the present situation of the stock markets, investment decision pressure, and family members’ psychological health now put pressure on investor psychology.

### Investors’ Psychology (Sentiments), Stock Market, and COVID-19

**FIGURE 1:**
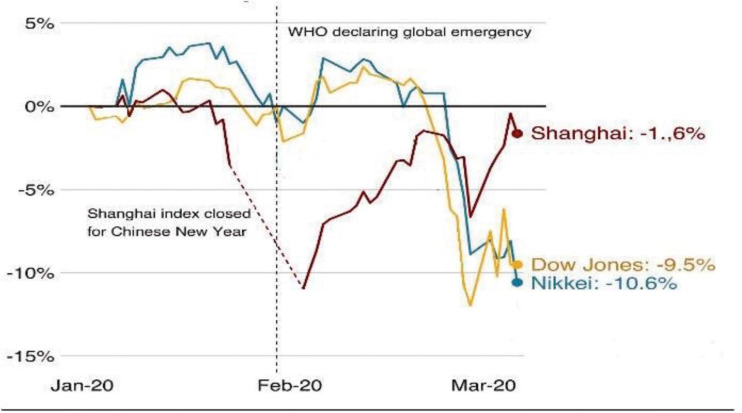
Impact of COVID-19 on stock markets: Source Bloomberg.

The COVID-19 outbreak has threatened every individual field of life to influence public health. The sustainability of the global stock market and financial markets also carries significant repercussions (Ali et al., 2020; Huang and Zheng, 2020). Being a part of the societal system, investor psychology (sentiments) and their optimism or pessimism about future stock prices can also change. A sharp decrease has been observed in Shanghai, Dow Jones, and Nikkei’s stock prices due to investor sentiment volatility during the pandemic outbreak (see Figure 1). The visual presentation of Figure 1 has shown a sudden downward trend in stock markets after the outbreak of the pandemic.

Existing literature has focused on the relationship between stock prices and investor sentiment. Lee et al. (2002) and Brown and Cliff (2004) explained that the past market returns are important sentiment determinants, while investor sentiment changes significantly correlated with the contemporary market return. The positive relationship between stock markets and sentiment will confirm that investor sentiment is a contrarian predictor for consequent market returns. Meanwhile, the sentiment impact is stronger if easy (hard) to value stocks are negatively (positively) influenced by sentiments (Baker and Wurgler, 2007; Xiang et al., 2020). Using the component of market index return, which is avoidant of fundamental macroeconomic factors, Lan et al. (2020) observed robust evidence that the pre-announcement abnormal return derives from investor sentiment. The sentiment determined overvaluation corrects within 1 month in the post-announcement period. The market timers tackle this sentiment situation and take advantage of issuing season shares. The stock price sensitivity in terms of the good news of earning is higher during a high sentiment period. In contrast, in a low sentiment period, the stock price sensitivity behaves negatively. As per analysis suggestions, the investor sentiment becomes the reason for the general mispricing of stock because of sentient-driven mispricing of earning contributions (Schmeling, 2009; Zouaoui et al., 2011; Mian and Sankaraguruswamy, 2012; Cheema et al., 2020). The high market competition indicated that sentiments and returns are positively related to each other, and this relationship disappears in low market competition. Although the financial crisis changes the situation irrespective of market competition, a positive relationship exists between sentiments and returns (Ryu et al., 2020). Investors can accept psychological pressure more sensitively and intensively than the lay person. Apart from the pandemic’s rapid spread, the financial news, media, and amplifiers have worked as fear spreaders about COVID-19. Tetlock (2007) elucidated that spread of news about the stock market strongly affects investor psychology and sociology. The high media pessimism leads to downward pressure on market prices and vice versa. The investor sentiment theory also confirmed the consistent relationship between media content and individual investor behavior with disproportionately small stocks. This research is based on a new ideology of investor psychology and the stock market during the pandemic. There have been few studies in this area, but a bulk of research centers on both human psychology and COVID-19 as well as the stock market and COVID-19. Under the caption of investor psychology, the stock market, and COVID-19, we tried to explain this research’s nature and relationship.

The psychological pressure negatively impacts investors and investing decisions, which can decline any individual country’s economy. This study analyzed investor psychology and stock market behavior during COVID-19—a comparatively new debate about COVID-19. This research will contribute to the existing literature and open up new dimensions in understanding investor sentiment toward investment decisions in the stock market under special circumstances during the outbreak of pandemics and times of intense anxiety. Our research differs from previous studies in the use of proxies of investor sentiment as indicators of the stock market and COVID-19. The strong theoretical upbringing of psychological behavior and the dynamic process of stock price fluctuation will deepen the understanding of readers, investors, and researchers. A sample of three different stock markets will help elaborate on investors’ psychological and geographical sensation during investment decisions in a pandemic.

## Data Description and Methodology

### Data Description

Our research includes daily observations of three different stock markets, i.e., Shanghai stock market, Nikkei, and Dow Jones, from January 20 to April 27, 2020. The market selection is based on two reasons. The first is the impact of COVID-19 on investor sentiment during the pandemic, i.e., the Shanghai Stock Market (China). The second is to check the global impact by using Nikkei and Dow Jones. The reason behind the selected data span is the global spread of COVID-19. The sample period starts from the data declaration of all sample markets because the synchronized data lead to accurate results. The data are collected from stock markets in China, Japan, and the United States. The analyzed data are secondary and publically available on mentioned databases, i.e., Bloomberg for stock markets data and WHO for COVID-19.

### Methodology

The Sentiment Index (SMI) model used in this research is presented below:

(1) $$S\,M\,I_{m,t,i}=\alpha_{1}\,S\,T\,U\,R\,N+\alpha_{2}\,M\,F\,I+\alpha_{3}\,R\,S\,I+\alpha_{4}\,\Delta \,C\,C+\alpha_{5}\,\Delta \,C\,D+\varepsilon_{t}$$

In eq. 1, SMI_*m,t*_ indicates the first principal component estimated by eq. 1’s linear combination of the standardized variables. Stock exchange turnover ratio (STURN) is the turnover of the respective stock exchange, MFI is the Money Flow Index, RSI is the Relative Strength Index, Δ CC is the change in daily confirm cases, and Δ CD is the daily confirmed deaths.

#### Stock Exchange Turnover Ratio

The stock market’s trading activity can be measured by turnover ratio; subsequently, it is used in the primary measurement model. Ying (1966) and Rehman et al. (2017) have explained that more considerable turnover is an indication of a rise in stock prices (Bullish Market), while small turnover reflects a fall in stock prices (Bearish Market). The stock exchange turnover ratio is calculated by using the following equation:

(2) $$S\,T\,U\,R\,N=100\times \frac{V\,M_{D\,a\,i\,l\,y}}{V\,M_{M\,o\,n\,t\,h\,l\,y}}$$

where VM_*Daily*_ is used for daily volume, VM_*Monthly*_ is the average volume of the month, and STURN is calculated using a running or moving basis, which means the previous dropping value and adding the next one.

#### Money Flow Index

The MFI comprises daily stock prices and turnover information. An increase in money flow indicates the market trend. The rising trend in the MFI increases the buying pressure, whereas the rise in the falling trend increases the selling pressure. The following formula is used to calculate the MFI:

(3) $$D\,a\,i\,l\,y\,P\,r\,i\,c\,e\,s=\frac{L\,o\,w+H\,i\,g\,h+C\,l\,o\,s\,e}{3}$$

(4) M⁢o⁢n⁢e⁢y⁢F⁢l⁢o⁢w=D⁢a⁢i⁢l⁢y⁢P⁢r⁢i⁢c⁢e⁢s×T⁢u⁢r⁢n⁢o⁢v⁢e⁢r

When the current day price is higher than the previous day, the money flow is positive, while there is a comparatively lower current day price than the previous day, the money flow is negative (Tolonen, 2011; Wang et al., 2015; Marek and Marková, 2020). The daily MFI has been calculated as follows:

(5) $$M\,F\,I=100\times \frac{P\,o\,s\,i\,t\,i\,v\,e\,M\,o\,n\,e\,y\,F\,l\,o\,w_{D\,a\,i\,l\,y}}{P\,o\,s\,i\,t\,i\,v\,e\,M\,o\,n\,e\,y\,F\,l\,o\,w_{D\,a\,i\,l\,y}+N\,e\,g\,a\,t\,i\,v\,e\,M\,o\,n\,e\,y\,F\,l\,o\,w_{D\,a\,i\,l\,y}}$$

#### Relative Strength Index

The technical analysis used the RSI, a momentum indicator that measures the magnitude of recent price changes, to evaluate the oversold or overbought condition in stock or other asset prices (Russell, 1978; Wilder, 1978; Russell and Franzmann, 1979; Ivascu and Cioca, 2019). An oscillator is a display board of RSI between two extremes (low and high with a range of 0–100). Suppose the oscillator shows an upward trend with RSI ≥ 70 value, meaning that a security is overbought or overvalued. In that case, a positive but downward trend with RSI ≤ 30 value indicates an oversold or undervalued condition.

(6) $$R\,S\,I_{D\,a\,i\,l\,y}=100\times \frac{\Sigma \,{\left(P_{t,i}-P_{t-1,i}\right)}_{+}}{\Sigma \,\left|P_{t,i}-P_{t-1,i}\right|}$$

$${\left(P_{t,i}-P_{t-1,i}\right)}_{+}=\left|P_{t,i}-P_{t-1,i}\right|i\,f\,P_{t,i}-P_{t-1,i}>0,o\,t\,h\,e\,r\,w\,i\,s\,e\,P_{t,i}-P_{t-1,i}=0$$

#### Change in Daily Confirmed and Death Cases

The changes in daily confirmed and death cases of COVID-19 are used to capture investor mood swings regarding the spreading pandemic. This term was used by Chen et al. (2010, 2014) to check the impact of market index change on investor mood. The changes in daily confirmed cases and daily death cases are calculated as follows:

(7) $$\Delta \,C\,C=C\,C_{t}-C\,C_{t-1,i}$$

(8) $$\Delta \,C\,D=C\,D_{t}-C\,D_{t-1,i}$$

#### Relationship Between Stock Market Index and Investor Sentiment

The SMI regressed on the stock market volatility series during COVID-19. The following regression equation checks the respective sentiment and market return relationship:

(9) $$Y_{m,t-1,i}=\alpha +\beta \,l\,n\,S\,M\,I_{m,t-1,i}$$

*Y*_*m,t–1*_ is the market return of the stock market’s indicator concerning time, while SMI_*m,t–1,i*_ is the Sentiment Index. The calculation of *Y*_*m,t–1*_ is done by the following equation:

(10) $$R_{m,t,i}=100*L\,o\,g\,\left(\frac{P_{t}}{P_{t-1}}\right)$$

In this equation, *P*_*t*_ is the current market price (closing), and *P*_*t–1*_ is the preceding market price (closing).

## Results

### Principle Component Analysis

The principle component analysis (PCA) is employed to extract meaningful information from multivariate data (orthogonal linear transformation) and present the information in the form of a set of new variables by use of scalar projections, which are called principal components (PC). The total number of PCs is less than or equal to the original number of variables. That is why the new variables or PCs are known as a linear combination of actual variables. The PCs are used as direction identifiers and correspond to the total variation of the data set. The multivariate data dimensionality reduces by using PCA with minimal loss of information. The eigenvalues explained that every PC retains the amount of variation. The division of variation between PCs as the eigenvalues is large for first PCs and small for subsequent ones. The first PC, or the PC with more than one eigenvalue, was used to check the correlation because of the increased variation retention of the data set.

### Shanghai Stock Market

The principal component analysis of the selected variable for the Shanghai Stock Market is presented in Tables 1, 2. According to Kaiser Criterion, the principle component with an eigenvalue not less than 1 will be used (Yeomans and Golder, 1982; Braeken and Van Assen, 2017; Rehman et al., 2017). The eigenvalue of PC-1 is 2.0545, which meets the criteria of maximal variation. The numeric presentation of PC-1 shows 41.09% of the Shanghai Stock Market relationship, which is the highest value compared to other principal components. The following index was created by using the first principle component:

**TABLE 1:** Principle component analysis (PCA) of the variables (China).

The Eigenvalues	Number	Value	Difference	Proportion	Cumulative value	Cumulative proportion
Eigenvalues: (Sum = 5, Average = 1)	1	2.0545	0.4404	0.4109	2.0545	0.4109
	2	1.6141	0.7071	0.3228	3.6686	0.7337
	3	0.9070	0.5014	0.1814	4.5757	0.9151
	4	0.4057	0.3870	0.0811	4.9813	0.9963
	5	0.0187	–	0.0037	5	1

*Source: Author’s calculation.*

**TABLE 2:** The eigen vector loadings (Japan).

Variable	PC 1	PC 2	PC 3	PC 4	PC 5
STURN	−0.0584	0.6588	−0.3581	0.6589	−0.0114
MFI	0.1759	0.6664	−0.1181	−0.7143	0.0283
RSI	−0.1244	0.3472	0.9187	0.1411	−0.0067
ΔCC	0.6881	−0.0323	0.0870	0.1528	0.7033
ΔCD	0.6904	−0.0193	0.0786	0.1109	−0.7102

*Source: Author’s calculation.*

$$SMI_{S\,S\,E,t}=-0.0584STURN+0.1759MFI+-0.1244RSI+0.6881\,\Delta \,C\,C+0.6904\,\Delta \,C\,D$$

**FIGURE 2:**
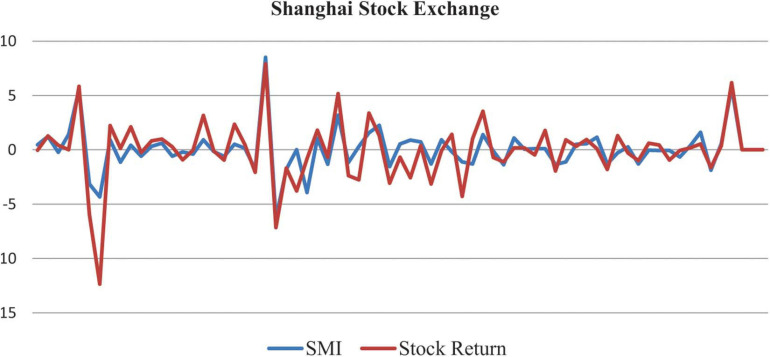
Relationship between Shanghai stock returns and created Sentiment Index.

The relationship between Shanghai stock returns and the created SMI is graphically presented in Figure 2.

The correlation matrix results are displayed in Table 3, which is employed to check the multicollinearity among independent variables. The multicollinearity check is essential for the accuracy of results because inter-correlation among independent variables in a multiple regression model can mislead the results. When the regressor shows a value of more than 0.80, then the data series shows multicollinearity. The correlation matrix range for the Shanghai Stock Market is from −0.0991 to 0.8018, ensuring the data series is free from multicollinearity.

**TABLE 3:** Ordinary correlations (United States).

	STURN	MFI	RSI	ΔCC	ΔCD
STURN	1				
MFI	0.5348	1			
RSI	0.1233	0.1892	1		
ΔCC	−0.1045	0.1607	−0.1127	1	
ΔCD	−0.0991	0.1878	−0.1153	0.8018	1

*Source: Author’s calculation.*

#### Nikkei 225 Stock Market

The PCA of the selected variable for the Nikkei 225 stock market is presented in Tables 4, 5. According to Kaiser Criterion, the principle component with an eigenvalue not less than 1 will be used (Yeomans and Golder, 1982; Braeken and Van Assen, 2017; Rehman et al., 2017). The eigenvalue of PC-1 for the Nikkei 225 stock market is 1.8735, which captures maximum variation and gets the full support of Kaiser Criterion. The cumulative proportion value of PC-1 shows 37.47% of the Nikkei 225 stock market relationship with a set of selected variables. The following index was created by using the first principle component:

**TABLE 4:** Principle component analysis (PCA) of the variables (China).

The eigenvalues	Number	Value	Difference	Proportion	Cumulative value	Cumulative proportion
Eigenvalues: (Sum = 5, Average = 1)	1	1.8735	0.6057	0.3747	1.8735	0.3747
	2	1.2678	0.3853	0.2536	3.1413	0.6283
	3	0.8825	0.0721	0.1765	4.0238	0.8048
	4	0.8104	0.6446	0.1621	4.8342	0.9668
	5	0.1658	–	0.0332	5	1

*Source: Author’s calculation.*

**TABLE 5:** The eigen vector loadings (Japan).

Variable	PC 1	PC 2	PC 3	PC 4	PC 5
STURN	0.6844	−0.1151	−0.1598	−0.0468	0.7005
MFI	−0.1786	0.6863	0.3105	−0.5451	0.3217
RSI	−0.6003	−0.3142	0.2582	0.3109	0.6145
ΔCC	−0.1380	0.5865	−0.5755	0.5360	0.1357
ΔCD	0.3469	0.2702	0.6929	0.5628	−0.0989

*Source: Author’s calculation.*

$$SMI_{N\,225,t}=0.6844STURN+-0.1786MFI+-0.6003RSI+-0.1380\Delta CC+0.3469\Delta CD$$

**FIGURE 3:**
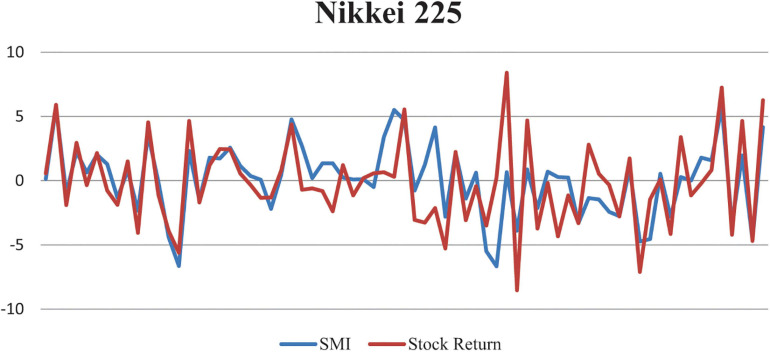
Relationship between Nikkei 225 market returns and created Sentiment Index.

The relationship of Shanghai stock returns and created SMI is graphically presented in Figure 3.

The correlation matrix results are displayed in Table 6, which is employed to check the multicollinearity among independent variables. The range of correlation matrix for the Nikkei 225 stock market is between −0.0991 and 0.8008, which rejects the existence of multicollinearity.

**TABLE 6:** Ordinary correlations (United States).

	STURN	MFI	RSI	ΔCC	ΔCD
STURN	1				
MFI	0.5349	1			
RSI	0.1234	0.1892	1		
ΔCC	−0.1045	0.1607	−0.1128	1	
ΔCD	−0.0991	0.1878	−0.1154	0.8008	1

*Source: Author’s calculation.*

### Dow Jones Stock Market

The PCA of the selected variable for the Dow Jones stock market is presented in Tables 7, 8. The eigenvalue of PC-1 is 1.7291, which captures maximum variation and gets the full support of Kaiser Criterion (Yeomans and Golder, 1982; Cioca et al., 2014; Braeken and Van Assen, 2017; Rehman et al., 2017). The cumulative proportion value of PC-1 shows 34.58% of the Dow Jones stock market relationship with chosen variables, which is the highest value among all principal components. The following index was created using the first principle component:

**TABLE 7:** Principle component analysis (PCA) of the variables (China).

	Number	Value	Difference	Proportion	Cumulative value	Cumulative proportion
**The eigenvalues**						
Eigenvalues: (Sum = 5, Average = 1)	1	1.7291	0.2446	0.3458	1.7291	0.3458
	2	1.4845	0.7252	0.2969	3.2136	0.6427
	3	0.7594	0.0917	0.1519	3.9730	0.7946
	4	0.6677	0.3083	0.1335	4.6406	0.9281
	5	0.3594	–	0.0719	5	1

*Source: Author’s calculation.*

**TABLE 8:** The eigen vector loadings (Japan).

Variable	PC 1	PC 2	PC 3	PC 4	PC 5
STURN	−0.2275	0.5515	0.6948	0.3344	0.2226
MFI	−0.1650	0.5628	−0.6981	0.4107	−0.0009
RSI	−0.1960	−0.5985	0.0193	0.7743	0.0590
ΔCC	0.6798	0.0110	−0.0776	0.1278	0.7179
ΔCD	0.6484	0.1443	0.1534	0.3219	−0.6570

*Source: Author’s calculation.*

$$SMI_{D\,J,t}=-0.2275STURN+-0.1650MFI-0.1960RSI+0.6798\,\Delta \,C\,C+0.6484\,\Delta \,C\,D$$

**FIGURE 4:**
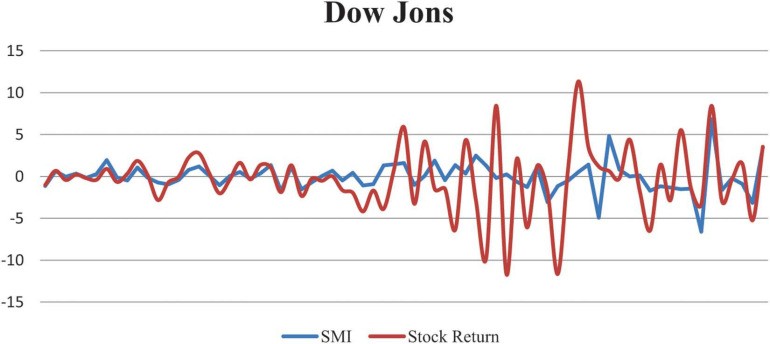
Relationship between Dow Jones stock market returns and created Sentiment Index.

The relationship between Dow Jones stock returns and the created SMI is graphically presented in Figure 4.

The correlation matrix results are displayed in Table 9, which is employed to check the multicollinearity among independent variables. The importance of multicollinearity is observed because inter-correlation among independent variables in a multiple regression model can betray the results. The range of the correlation matrix for the Dow Jones stock market is from −0.0367 to 0.6135, which guarantees the data series free from multicollinearity.

**TABLE 9:** Ordinary correlations (United States).

	STURN	MFI	RSI	ΔCC	ΔCD
STURN	1				
MFI	0.2490	1			
RSI	−0.2251	−0.2420	1		
ΔCC	−0.2134	−0.1088	−0.1600	1	
ΔCD	−0.0367	−0.0572	−0.1932	0.6135	1

*Source: Author’s calculation.*

The regression results are presented in Table 10, which shows the coefficient (β) −0.2532, −0.2532, and −0.0264 as the value of the SMI with 0.0056, 0.0056, and 0.0000 probability values for Shanghai, Nikkei 225, and Dow Jones stock markets, respectively. According to this study, the SMI is negative and significantly related to stock returns at a 1% level of significance (Baker and Wurgler, 2007; Chen et al., 2014). The importance of SMI explicated that investor sentiments are strongly affected by volatility and investment decision of the stock market during the pandemic. The results have also shown the negative impact of COVID-19 on investor sentiment and stock market returns. The spreading pandemic disturbs the general public’s daily routines and interrupts stock markets, financial markets, and investor psychology toward investment decisions.

**TABLE 10:** The relationship between stock exchange and Sentiment Index during COVID-19.

	α (consent)	β
**Shanghai stock exchange**		
SMI (Sentiment Index)	−0.9453	−0.2532*
Prob.	0.0079	0.0056
*T*-statistic	[−2.7388]	[−2.8627]
**Nikkei 225**		
SMI (Sentiment Index)	−0.9453	−0.2532*
Prob.	0.0079	0.0056
*T*-statistic	[−2.7388]	[−2.8627]
**Dow Jones**		
SMI (Sentiment Index)	8.6546	−0.0264*
Prob.	0.0000	0.0000
*T*-statistic	[117.3315]	[−6.5732]

*Shows 1% level of significance.

*Source: Author’s calculation.*

## Discussion

After the pandemic outbreak and the WHO’s classification of it as a public health emergency, investors’ psychological pressure and response showed a downward trend in stock markets. The sudden reduction in Shanghai (1.6%), Dow and Jones (9.5%), and Nikkei (10.6%) can be observed in Figure 1. The numerical facts of stock markets are collected from Bloomberg’s official site, and the investor sentiment is generated using different proxies. The selected proxies represent different circumstances that might be affected the psychological behavior and decision power of investors. In this research, daily data of stock markets and increase in COVID-19 (death and confirmed cases) are used, accurately covering investors’ daily psychological pressure. The PCA is employed due to its useful features: correlation removal, improved algorithm performance, repaired overfitting among variables, and reduction of high dimensions into low dimensions for clear visualization of every single component. The research results elucidated a negative and significant relationship between investor psychology and investment decision under pandemic outbreaks for all selected markets. The stock market movement along the investor SMI in Figures 2–4 has shown the beneficial relationship between investor psychology and stock market returns. The investors or business people were generally outbound for 10–15 h per day. During pandemic, however, they were homebound, which affected their psychology adversely. This research provides some precautionary measures for releasing the pandemic and investment pressure. Investors should adopt behavior therapy—home-based relaxation exercises to control their anxiety and depression. The small-scale version of their official stock market setup should be established in their homes, and visits to the offices should be reduced. The global paramedical staff and scientists are continually struggling to elucidate the vaccines. Until they succeed, everyone should follow the precautions, i.e., wearing a mask, sanitizing, and maintaining distance in workplaces and the like. The less psychological control or pressure can help investors invest money and keep stock markets and economies on track.

## Conclusion

The origin of the current “COVID-19” pandemic is considered to be the wet market of Hunan, Hubei Province, China. Within 1 month from its evolution, COVID-19 has spread to 109 countries, and the pandemic has gained intense global attention. The sudden outbreak of the pandemic and the rapid increase of its spread have left a significant impact on human physiology and psychology. The psychological effect disrupts the psychology of the general public and investor psychology toward stock market investment decisions. The increasing number of cases and deaths worldwide due to COVID-19 has made the economic situation more uncertain and unpredictable. A sudden and dramatic downward trend in financial markets is observed in Chinese and global financial markets, such as Shanghai, Nikkei 225, and Dow Jones, which are down by −1.6, 10.6, and −9.5% points, respectively. There are no promising clinical treatments or prevention strategies developed against COVID-19 until now, threatening human psychology. At the same time, healthcare workers are searching for a solution to the question of vaccination against COVID-19 and psychiatrist-designed psycho-therapeutic strategies to cope with the threat, stress, and anxiety of the pandemic, which have a devastating effect on daily life.

This research paper examined the relationship between the stock market and investor psychology regarding stock market investment decisions during the pandemic. By employing PCA, this research observed a downward trend in stock markets and the pandemic’s negative impact on investor sentiment. This investigation confirmed the economic crises in the Shanghai, Nikkei 225, and Dow Jones stock markets during the pandemic. The results have pointed out that the threat of health strongly affected the psychology of investors. The created SMI behaved negatively with a significance of 1% for three selected markets. The three selected markets represented three different world areas with diverse geographical backgrounds, financial positions, cultures, resources, and traditions to check global investor behavior. The significant relationship between the SMI and the stock market during a pandemic confirmed that the behavior of almost every nation fighting COVID-19 and investor financial behavior is the same across China and other developed countries. This study concluded that health crises and psychological disorders among the general public affect the economic condition and financial position of individual and global investors.

### Limitations and Suggestions

The pandemic is still under discussion, and healthcare workers are trying to find a solution to the issue of vaccination. It is doubtlessly tough to run global systems, such as the stock market, from the workers’ individual homes, but to stop working due to anxiety or psychological threat is also not the solution to the problem. Investor sentiment creates tremendous uncertainty for stock markets and commensurate with a potential crisis of scale and speed. The governments and policy-makers should have to set some domestic and international policies for this unpredictable situation for workplaces. The pandemic is a worldwide issue, but the courageous actions of governments, global citizens, policy-makers, healthcare workers, scientists, and investors can enable us to overcome this global crisis.

## Data Availability Statement

The raw data supporting the conclusions of this article will be made available by the authors, without undue reservation.

## Author Contributions

All authors listed have made a substantial, direct and intellectual contribution to the work, and approved it for publication.

## Conflict of Interest

The authors declare that the research was conducted in the absence of any commercial or financial relationships that could be construed as a potential conflict of interest.
